# Insights into Triterpene Acids in Fermented Mycelia of Edible Fungus *Poria cocos* by a Comparative Study

**DOI:** 10.3390/molecules24071331

**Published:** 2019-04-04

**Authors:** Jian Jin, Rongrong Zhou, Jing Xie, Huixuan Ye, Xuejuan Liang, Can Zhong, Bingbing Shen, You Qin, Shuihan Zhang, Luqi Huang

**Affiliations:** 1 Institute of Chinese Materia Medica, Hunan Academy of Chinese Medicine, Changsha 410013, China; jinjian2016@163.com (J.J.); axxj2057@163.com (J.X.); guole18888@163.com (X.L.); canzhong651@163.com (C.Z.); shenbingbing24@126.com (B.S.); 20162010@stu.hnucm.edu.cn (Y.Q.); 2 College of Pharmacy, Changchun University of Chinese Medicine, Changchun 130117, China; rz172@georgetown.edu; 3 National Resource Center for Chinese Meteria Medica, Chinese Academy of Chinese Medical Sciences, Beijing 100700, China; 4 Jiuzhitang Co., Ltd, Changsha 410205, China; yehuixuan2019@163.com; 5 2011 Collaboration and Innovation Center for Digital Chinese Medicine in Hunan, Changsha 410208, China

**Keywords:** fungi, mycelium, sclerotium, triterpene, HPLC-QTOF-MS/MS

## Abstract

As an edible sclerotia-forming fungus, *Poria cocos* is widely used as a food supplement and as a tonic in China. High-performance liquid chromatography-quadrupole/time-of-flight mass spectrometry (HPLC-QTOF-MS/MS) was applied to identify triterpene acids in fermented mycelia of *P. cocos*, as well as the epidermis and inner part of natural sclerotia. A total of 19 triterpene acids were identified in fermented mycelia, whereas 31 were identified in the epidermis and 24 in the inner part. Nine triterpene acids were quantitatively determined, and the concentrations of two valuable triterpenes, dehydropachymic acid and pachymic acid, reached 1.07 mg/g and 0.61 mg/g in the fermented mycelia part, respectively, and were both significantly higher than the concentration in the two natural parts. The fermented mycelia could be a good choice for producing some target triterpene compounds and functional foods through fermentation thanks to the high concentration of some triterpene acids.

## 1. Introduction

*Poria cocos* (Schwein.) F.A. Wolf [1] is a well-known edible fungus with forming sclerotia, which contain the compact mass of hardened fungal mycelia containing food reserves [1]. This fungus is known as “Indian bread” in North America [2] and has also been used to make food supplements, Chinese meals, and healthy snacks and desserts in China and some other countries of East Asia [3]. This fungus is also a medicinal species, according to *Chinese Pharmacopoeia* (2015 edition). Two parts of sclerotia of *P. cocos* could be used as crude drugs for their health promotion benefits, i.e., the inner part (Baifuling in Chinese, or BFL) and the epidermis (Fulingpi in Chinese, or FLP).

Regarding chemical composition, *P. cocos* contains low phenol content and flavonoid content [4] but is a rich source of triterpene acids, which are generally considered one of the principal bioactive components. To date, more than 120 triterpenoid compounds have been identified from *P. cocos* [5]. These triterpene acids can be classified into 4 subgroups according to their chemical structure, i.e., lanostane-8-ene type (type I), lanostane-7,9(11)-diene type (type II), 3,4-seco-lanostane-8-ene type (type III) and 3,4-secolanostane-7,9(11)-diene type (type IV) compounds. Modern phytochemical and pharmacological investigations show that *P. cocos* has a lot of therapeutic potential, and the triterpene compounds in *P. cocos* are reported to be responsible for diuretic [6,7], anti-tumor [8], and anti-inflammatory activities [9,10].

As one species of brown-rot fungi, *P. cocos* grows as a saprophytic fungus on the stem or root of pine trees. As *P. cocos* is widely used in traditional Chinese medicine, the massive demand leads to a lot of coniferous trees being felled to cultivate this fungus, destroying the forest ecological environment. It is worth noting that the liquid fermentation of mycelia could be an alternative to producing *P. cocos*, just like the fermentation of Cordyceps fungus and Ganoderma [11,12]. The accumulation of the mycelia biomass of *P. cocos* through fermentation has been demonstrated to be possible [13,14]. However, to date, there are few reports on the comparison of the bioactive triterpene acids profile in natural sclerotia and in fermented mycelia of *P. cocos*. 

In this study, high-performance liquid chromatography-quadrupole/time-of-flight mass spectrometry (HPLC-QTOF-MS/MS) was applied to analyze the chemical profile of three kinds of samples, i.e., epidermis, inner part of the sclerotia and the fermented mycelia of *P. cocos*. To the best of our knowledge, this study represents the first report of comparative identification and quantification of triterpene acids in different forms of *P. cocos*, and it will be of value for understanding the potential for better use of fermented mycelia.

## 2. Results

### 2.1. The Natural and Fermented Parts of Poria Cocos

**Figure 1:**
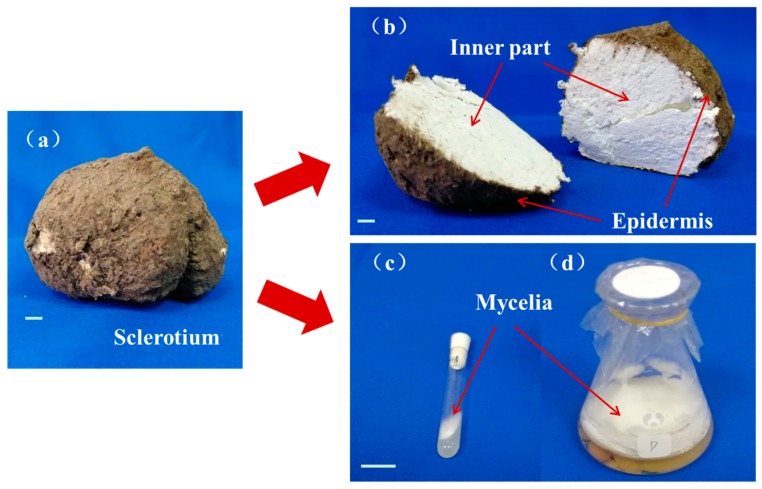
Whole sclerotium (**a**), cut sclerotium of *Poria cocos* (**b**), isolated *P. cocos* strain kept in a glass tube (**c**) and mycelia fermented in liquid medium (**d**); bar, 2 cm.

*P. cocos* is a well-known saprophytic fungus, which could grow into sclerotia in the natural environment (Figure 1a,b).

The white, dense inner part of the sclerotium, known as Fuling or Baifuling in Chinese, is widely used as traditional Chinese medicine and as functional food in many countries [1]. *Poria cutis*, epidermis of the sclerotium, named Fulingpi in Chinese, is another medicinal material as recorded in *Chinese Pharmacopoeia*. A two-stage fermentation combining shaking fermentation and static culture was carried out to obtain the mycelia (Figure 1c), which is a strategy often applied for enhancing secondary metabolite production by medicinal fungus [15]. 

### 2.2. Identification of Triterpene Acids in Mycelia, Epidermis and the Inner Part

**Figure 2:**
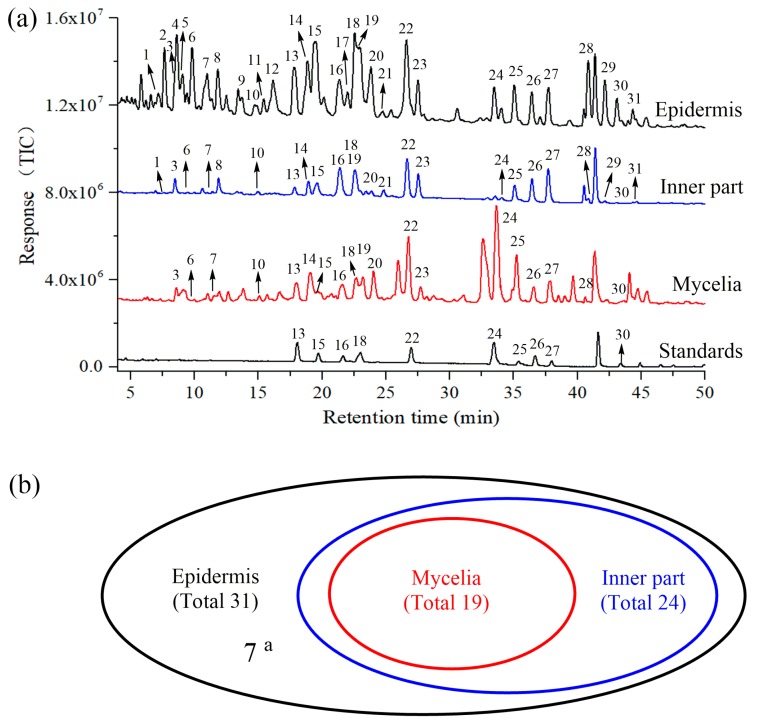
Total ion chromatography (TIC) of epidermis, inner part and mycelia of *P. cocos* and standards of triterpene acids (**a**) and Venn diagram of the number of identified triterpene acids in epidermis, inner part and mycelia of *P. cocos* (**b**); ^a^, the number of triterpene acids only identified in epidermis.

The chemical profiles of dissected parts were comprehensively analyzed through HPLC-QTOF-MS/MS in negative ion mode, because more stable and repeatable ions of triterpene compounds could be provided in negative ion mode than in positive ion mode [16]. The representative LC-MS total ion chromatograms for epidermis, inner part and mycelia of *P. cocos* are shown in Figure 2a. A total of 31 chromatographic peaks were characterized on the basis of retention time, accurate molecular mass, generated molecular ions and fragment ions by matching this data with corresponding data of known compounds in our in-house library. Ten of these peaks, namely, pachymic acid (PA), dehydropachymic acid (DPA), 3-*O*-acetyl-16*α*-hydroxytrametenolic acid (AHTRA), dehydrotumulosic acid (DTUA), 16*α*-hydroxydehydrotrametenolic acid (HDTRA), dehydroeburicoic acid (DEA), 3-*O*-acetyl-16*α*-hydroxydehydrotrametenolic acid (AHDTRA), polyporenic acid C (PAC), poricoic acid A (PAA) and poricoic acid B (PAB), were unequivocally identified by comparing the retention time and mass data of reference standards.

**Figure 3:**
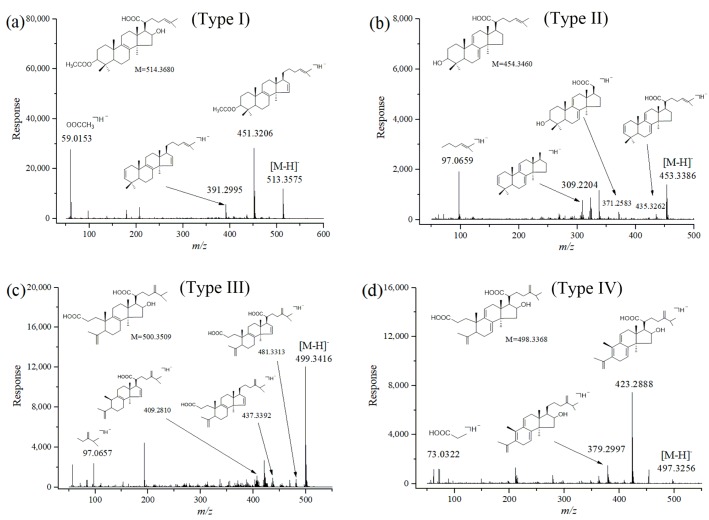
The MS/MS spectrum of 3-*O*-acetyl-16*α*-hydroxytrametenolic acid (**a**), dehydrotrametenolic acid (**b**), poricoic acid H (**c**) and poricoic acid A (**d**) identified by HPLC–QTOF–MS/MS.

The typical mass spectra and proposed fragmentation pathways of the four types of triterpenoid acids, namely, AHTRA (Type I), dehydrotrametenolic acid (Type II), poricoic acid H (Type III) and PAA (Type IV), are given in Figure 3. The major pathways for the four types of triterpenoid acids contained: characteristic neutral losses of 18 Da (H_2_O), 44 Da (CO_2_), and 74 Da (CH_3_CH_2_COOH); and typical cleavage of side chains at the C-3 or C-20 position. For example, peak 26 showed [M − H] ^−^ at m/z 513.3575 in the negative ion mode, indicating that its molecular formula was C_32_H_50_O_5_. In the MS/MS spectrum, fragment ions at m/z 493.3261, m/z 451.3206 and m/z 391.2995 generated by sequential losses of H_2_O, CO_2_, and the CH_3_COOH side chain at C-3 position were clearly observed. Therefore, peak 26 was identified as AHTRA [3,16,17,18], a lanostane-8-ene-type triterpene (type I), which was further confirmed by comparison with the reference compound. Similarly, the molecular formula of peak 29 was characterized as C_30_H_46_O_3_ based on its mass-to-charge ratio of m/z 453.3386 ([M − H]^−^). In addition, dehydrated product ions at m/z 435.3262 ([M–H − H_2_O]^−^), m/z 371.2583 ([M − C_6_H_11_]^−^) and m/z 309.2204 were formed by eliminating C_6_H_11_ (83 Da) side chain at the C-20 position, and by further eliminating H_2_O, CO_2_. Peak 29 was thus assigned as dehydrotrametenolic acid (a lanostane-7,9(11)-diene type triterpene, type II) [3,16,17,18]. A precursor ion with an m/z value of 499.3436 [M − H]^−^ was observed at peak 7 in the full scan spectrum. This ion afforded the fragment ions with an m/z value of 481.3313, 437.3392 and 409.2810 as the base peak in the MS/MS spectrum. These ions were attributed to the loss of H_2_O, CO_2_ and the CH_3_CH_2_COOH side chain. The transitions of these ions were similar in many ways to those reported in the literature [3,16,17,18], and they were finally identified as poricoic acid H (type III, 3,4-seco-lanostane-8-enes triterpene). The fragmentation behavior of PAA was analyzed in detail as a good example of type IV 3,4-seco-lanostane-7,9(11)-diene triterpene compound. The full scan spectrum of PAA contained a precursor ion with an m/z value of 497.3256 [M–H]^−^, characterized as C_31_H_46_O_5_. Fragment ions with value of 423.2888 and 379.2997 were observed in the MS/MS spectrum. These fragments showed the neutral loss of 74 Da (m/z 497 → 423) and 118 Da (m/z 497 → 379), which were consistent with the loss of the CH_3_CH_2_COOH moiety and successive loss of CO_2_. Similar results were obtained to those reported before [3,17,18]. Overall, these characteristics of neutral losses and cleavage of side chains provide sound support for identification of the triterpene acids. 

The other reference compounds and identified triterpenes were analyzed as well, and their characteristic ions are shown in Supplementary Table S1. In total, there were 31 triterpene acids identified in epidermis but only 24 in the inner part and 19 in mycelia of *P. cocos* (Figure 2b). Specifically, the triterpene acids identified in mycelia were covered by that in the inner part and epidermis. 

### 2.3. Quantitative Analysis of Nine Triterpene Acids in Mycelia, Epidermis and the Inner Part

Nine triterpene acids, namely, PA, DPA, AHTRA, DTUA, HDTRA, DEA, AHDTRA, PAC and PAB, were selected as reference standards for quantitation. The calibration curves showed good linearity with R^2^ ≥ 0.99 for all the nine triterpene acids in a certain concentration range (Table 1). The limit of detection (LOD) and limit of quantification (LOQ) were in the range of 0.016–0.350 μg/mL and 0.054–1.166 μg/mL, respectively. The intraday and interday variations (RSDs) of the compounds were within 1.42–3.21% and 2.38–4.151%, respectively. The RSDs for stability were lower than 4.81%. The average recoveries ranged from 90.25% to 96.16%, and their RSDs were within 9.42% (Table 2). All these parameters demonstrated that the quantitative method was linear, precise, stable, sensitive and accurate enough for the determination of main triterpene acids. 

**Figure 4:**
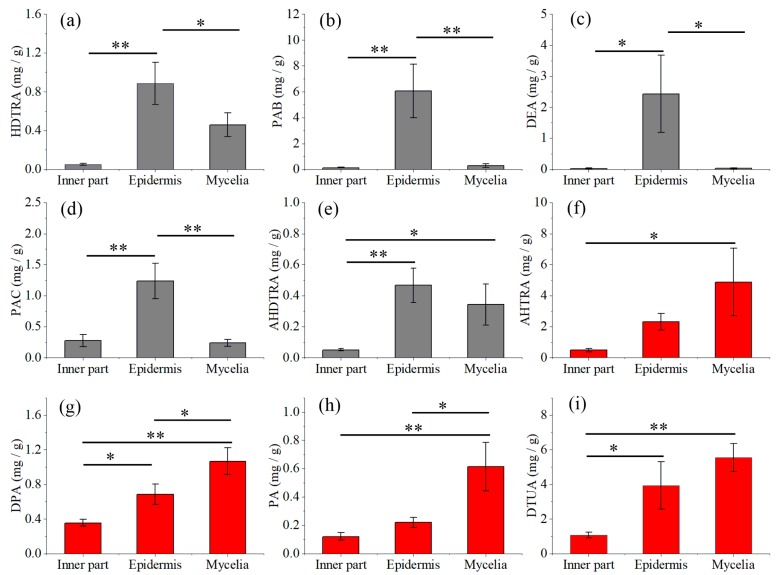
Quantitative comparison of the content of triterpene acids HDTRA (**a**), PAB (**b**), DEA (**c**), PAC (**d**), AHDTRA (**e**), AHTRA (**f**), DPA (**g**), PA (**h**) and DTUA (**i**) in the inner part, epidermis and mycelia of *P. cocos*; data are mean ± SD (* *p* < 0.05, ** *p* < 0.01).

The contents of these nine compounds in natural and fermented samples of *P. cocos* are shown in Figure 4. The contents of HDTRA, PAB, DEA, PAC and AHDTRA were higher in epidermis than those in the inner part and in mycelia. The mycelia contained higher concentrations of AHTRA, DPA, PA and DTUA, compared with the inner part and epidermis of natural sclerotia. The concentrations of DPA and PA reached 1.07 mg/g and 0.61 mg/g in the mycelia, respectively, and were significantly higher in the mycelia than in epidermis (*p* < 0.05) and the inner part (*p* < 0.01). 

### 2.4. Principal Component Analysis of Mycelia, Epidermis and the Inner Part

**Figure 5:**
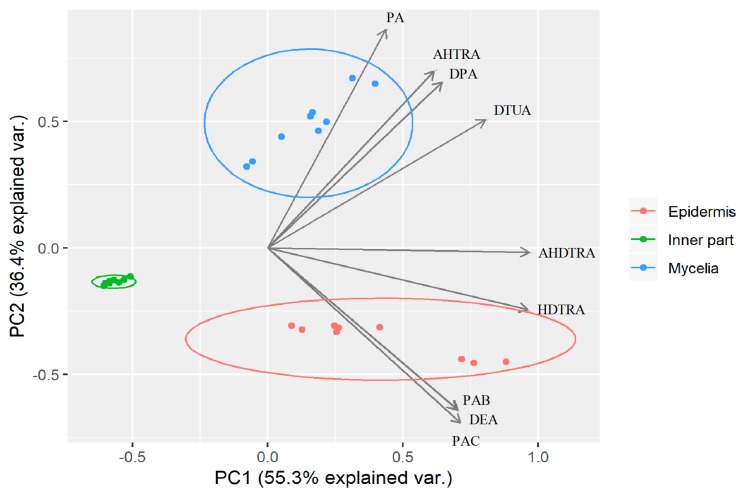
Biplot showing different samples of epidermis, inner part and mycelia of *P. cocos* on a two-dimensional space derived from principal component analysis (PCA) based on triterpene acids; the arrows indicate the projections of the original features onto the principal components.

The contents of PA, DPA, AHTRA, DTUA, HDTRA, DEA, AHDTRA, PAC and PAB in all the samples were considered as the variables of principal component analysis (PCA), as the triterpene acids are normally used as markers to evaluate the quality of *P. cocos*. The PCA biplot displayed the scores and loadings of the first two components (PC1 and PC2), revealing the projection of an observation on the subspace with score points (Figure 5). The variation in the pattern of quantified triterpene acid concentrations (55.3% and 36.4%) were explained by PC1 and PC2, respectively. These two components together explained 91.7% of the variation. The variables of AHDTRA, HDTRA, and DTUA shown in the same direction correlated with PC1. Pachymic acid, AHTRA and DPA were primarily correlated with PC2, whereas PAB, DEA and PAC were oppositely correlated with PC2. Principal component analysis results could substantially separate epidermis, the inner part and mycelia into three groups. 

## 3. Discussion

The natural parts of *P. cocos* are the source of triterpene acids, which are responsible for various medical effects [19]. It is worth noting that this fungal species could be fermented in mycelia form as well, potentially offering an alternative to the eco-destructive conventional production. Nevertheless, previous studies were only carried out on the epidermis and inner part of the sclerotium [16,17,18,20], giving no information of the triterpene acids profile in the fermented mycelia which could be developed into functional food. 

Although this is the first time that triterpene acids have been characterized in mycelia of *P. cocos*, present results are basically consistent with a previous report that more triterpene acids could be identified in epidermis than other parts [16,18]. Considering that the sclerotia were cultivated on the stems or roots of pine trees, it could be speculated that more types of triterpene acids identified in natural parts result from the pine-dependent cultivation.

There are already some studies investigating the quantitative analysis of triterpenoids acids in *P. cocos*. For instance, eight major triterpenoid acids [18] and nine triterpene acids [16] have been quantitatively compared in different parts of sclerotium of *P. cocos*. Also, seven major triterpenoids in 40 batches [3] and nine triterpene acids in 25 batches of *P. cocos* samples [17] from different origins have been quantified. The quantitative data in the present study is in agreement with evidence showing that the epidermis generally contained higher amounts of triterpene acids compared with the inner part [16]. Interestingly, the mycelia of *P. cocos* contained higher concentrations of AHTRA, DPA, PA and DTUA than those in the inner part and epidermis of sclerotia. Pachymic acid, the well-known lanostane type triterpenoid biomark of *P. cocos*, possesses anti-emetic, anti-inflammatory, and anti-cancer properties, and is also useful for the treatment of sleep-disturbed subjects with insomnia [21]. Dehydropachymic acid could decrease bafilomycin A1 induced β-Amyloid accumulation in PC12 cells, highlighting its therapeutic potential for Alzheimer’s disease treatment [22]. Consequently, fermented mycelia with a high concentration of these two triterpene acids attracts interest for potential development. 

Principal component analysis results were consistent with previous observations that samples of different parts of sclerotium could be well grouped by multivariate analysis [16,18]. To a certain degree, it seems that the mycelia could not replace the inner part or epidermis of sclerotium, because no overlap of these samples was observed.

Normally, *P. cocos* grows underground around the roots of pine trees and needs a long cultivation period of about 8 months. More notably, conventional production used a large number of trees, destroying the forest system. In recent years, higher fungi have received increasing attention as they produce numerous bioactive secondary metabolites [15]. Fermentation is a feasible strategy with potential for increased production of bioactive components in a compact space and shorter time [23]. Some traditional Chinese medicinal fungi have been fermented and used as medicine, dietary supplements or tonic food [24]. For example, “Bailing Jiaonang”, the capsule of fermented mycelia of *Ophiocordyceps sinensis* is popular for treating pulmonary disease in China [25]. The artificial fermented *Cordyceps militaris* has been approved as a kind of new food resource by the Ministry of Health of PRC and can be used as raw material of functional food [26]. *Ganoderma lucidum* is used to produce ganoderic acids by submerged fermentation of mycelia [11]. Fermentation culture of *P. cocos* and dynamic accumulation of three main triterpenic acids in submerged cultivation have been investigated [27]. The present work represents the first time that the triterpene acids profile in mycelia have been reported. Most of the triterpene acids could be identified in fermented mycelia of *P. cocos* and some of these bioactive components even at a high concentration level in mycelia. Although it might not be an alternative to using the fermented part to replace the traditional used sclerotium parts, it could be a good decision to develop the fermented mycelia part to produce some target triterpene compounds for functional foods.

## 4. Materials and Methods

### 4.1. Chemicals and Reagents

Pachymic acid (CAS No. 29070-92-6), DPA (CAS No. 77012-31-8), AHTRA (CAS No. 168293-13-8), DTUA (CAS No. 6754-16-1), HDTRA (CAS No. 176390-66-2), DEA (CAS No. 6879-05-6), AHDTRA (CAS No. 168293-14-9), PAC (CAS No. 465-18-9), PAA (CAS No. 137551-38-3) and PAB (CAS No. 137551-39-4) were purchased from Chroma Biotechnology Co., Ltd (Chengdu, China). Acetonitrile (HPLC-grade) was purchased from Merck (Darmstadt, Germany). Ultrapure water was purchased from Wahaha (Changsha, Hunan). Other reagents were of analytical grade. 

### 4.2. Collection of Sclerotia of Poria cocos

Three independent samples of *P. cocos* sclerotia were collected from Taiyangping farm of Busky Pharmaceutical Co., Ltd, located in Jingzhou County, Hunan Province, China, between latitude 26°15’38〞N–26°47’35〞N and long 109°16′4〞E–109°56′36〞E. They were authenticated by Professor Zhaoming Xie and Hao Liu from Hunan Academy of Chinese Medicine. The inner part and epidermis of sclerotium were separated. The voucher specimens (No. HACM-2018-A-C) were deposited in the Department of Pharmaceutical Chemistry, Hunan Academy of Chinese Medicine.

### 4.3. Strain Isolation

The fungus strain was isolated from the collected sclerotia of *P. cocos*, as described before with minor modification [28]. Briefly, the sclerotia were washed with distilled water to remove the surface soil, followed by surface sterilization with 75% ethanol spray. After that, the epidermis of sclerotia was cut off, and a small piece of the inner part was put into a petri dish with PDA medium. When the mycelia grow up, the front part of mycelia were picked and subcultured into a new PDA petri dish. Repeat the above operation until the mycelia of isolated strain is microscopically observed uniformly and confirmed by ITS identification. Then, the isolated strain was deposited in the collection of Hunan Academy of Chinese Medicine (accession number HACM-PC-HN-2801). 

### 4.4. Mycelia Fermentation 

The isolated strain of *P. cocos* was maintained in glass petri dishes (diamenter 11 cm) with potato dextrose agar (PDA) medium, which was prepared by weighing out 300 g potato, 20 g dextrose and 20 g agar in 1 L distilled water. Then, a small piece of solid PDA medium with mycelia was inoculated into liquid medium for fermentation in Erlenmeyer flask. The liquid medium contained peptone (5.0 g/L), glucose (15.0 g/L), KH_2_PO_4_ (1.0 g/L), MgSO_4_∙7H_2_O (0.5 g/L), and yeast extract powder (2.0 g/L). Liquid culture was maintained in an incubator at 25 °C, shaking at 130 rpm for the first 20 days, and then being static for the following 20 days. Finally, the mycelia were obtained by filtration. 

### 4.5. Extraction of Triterpene Acids

The collected inner part, epidermis of sclerotium and the mycelia were dried at 60 °C to constant weight. Then, samples were ground by passing through a 50-mesh sieve. Powdered samples (1.0 g), were accurately weighed, and extracted with 10 mL methanol through ultrasonication (KM-500DB, 40 KHz) for 30 min. After that, the extracts were centrifuged (3000 g for 15 min) to separate the supernatant from residues. The supernatant was passed through 0.22 μm syringe filter for HPLC-QTOF-MS/MS analysis.

### 4.6. HPLC-QTOF-MS/MS Analysis

Chromatographic analysis was carried out on an Agilent 1290 liquid chromatography system coupled with QTOF-MS/MS, which was equipped with an electrospray interface (Agilent 6530, Agilent Technologies, Santa Clara, CA, USA), in accordance with our previous studies [29]. Acetonitrile and formic acid/water (0.1%) were used as mobile phase A and B, respectively. Solvent flow rate was 1.0 mL/min and the column (InertSustain C18 column, 5μm, 4.6 × 250 mm, GL Sciences Inc. Tokyo, Japan) performed at 25 °C. The solvent gradient was set as follows: 51% A during 0–5 min, 51–72% A during 5–30 min, 72–86% A during 30–37 min, 86–100% A during 37–47 min, and 100% A during 47–50 min. Fractions were monitored at 210 and 245 nm. The condition of QTOF was as follows: scan range 100–1000 m/z, drying gas (N_2_) flow rate, 8.0 L/min; drying gas temperature, 320 °C; sheath gas temperature, 320 °C; capillary voltage, 3.5 kV; fragmentor, 110 V; collision energy at 30, 40 and 50 eV. Data analysis was done by the soft Qualitative Analysis B.05.00. Triterpene acids were determined according to precursor ions, the fragment ions and retention time compared to those of the standard compounds and our in-house library.

### 4.7. Quantitative Characterization of Triterpene Acids

Standard solutions of 9 triterpene acids, i.e., PA, DPA, AHTRA, DTUA, HDTRA, DEA, AHDTRA, PAC and PAB, at a series of appropriate concentrations were prepared for the construction of calibration curves. For AHTRA and PA, the monitor wavelength was set at 210 nm, and for other compounds at 245 nm. Then, the calibration curves were constructed by plotting the peak area versus the concentration. Limit of detection and LOQ were calculated at 3-fold and 10-fold of the signal-to-noise (S/N) ratios, respectively. Six replicates of each standard solution were analyzed within the same day and additionally on 3 consecutive days for evaluating intraday and interday variation. The sample extract was analyzed at 0, 2, 4, 8, 12, 24, and 48 h at room temperature for stability assessment. Recovery was determined by adding an accurately measured amount of standard compounds to the sample [29].

### 4.8. Statistical Analysis

The quantitative determination of triterpene acids was done in triplicate and expressed as mean ± standard deviation. Differences among groups were analyzed statistically using one-way analysis of variance (ANOVA). Statistical analyses of different samples were performed using the R statistical software (https://www.r–project.org) [29]. The data were imported through R software and treated using ggbiplot package to perform PCA [30]. 

## 5. Conclusions

The mycelia of *P. cocos* could be obtained by a two-stage fermentation combining shaking and static culture. A total of 19 triterpene acids were identified in mycelia. Although it might not be possible to use the fermented mycelia to replace the traditional used sclerotium, it could be used for producing some target triterpene compounds for functional foods by fermentation. 

## Figures and Tables

**Table 1:** Linear regression, limit of detection (LOD) and limit of quantification (LOQ) of investigated triterpene acids.

Compounds	Range (μg/mL)	Regression Equation ^a^	R^2^	LOD (μg/mL)	LOQ (μg/mL)
HDTRA	7.2–143.3	y = 0.0463x + 0.2607	0.999	0.018	0.061
PAB	17.3–145.6	y = 0.0767x + 0.3884	0.999	0.031	0.103
DTUA	7.1–141.1	y = 0.1353x − 0.0356	0.998	0.056	0.187
PAC	6.6–133.3	y = 0.0529x + 0.1776	0.998	0.023	0.077
AHDTRA	5.1–102.2	y = 0.0540x + 0.3526	0.992	0.350	1.166
AHTRA	7.0–140.0	y = 0.2383x + 3.2067	0.999	0.020	0.067
DPA	6.7–135.6	y = 0.0535x − 0.488	0.997	0.024	0.081
PA	4.8–97.7	y = 0.0186x + 0.1617	0.999	0.016	0.054
DEA	6.4–128.9	y = 0.0474x + 30.2911	0.999	0.016	0.055

^a^, x: peak area, y: concentration of the analyte (μg/mL). HDTRA, 16α-hydroxydehydrotrametendic acid; PAB, poricoic acid B; DTUA, dehydrotumulosic acid; PAC, polyporenic acid C; AHDTRA, 3-*O*-acetyl-16α-hydroxydehydrotrametenolic acid; AHTRA, 3-*O*-acetyl-16α-hydroxytrametenolic acid; DPA, dehydropachymic acid; PA, pachymic acid; DEA, dehydroeburicoic acid.

**Table 2:** Precision, stability and recovery of investigated triterpene acids.

Compounds	Precision (*n* = 6)		Stability (48 h) (RSD, %)	Recovery (*n* = 3)	
	Intra-day (RSD, %)	Inter-day (RSD, %)		Mean (%)	RSD (%)
HDTRA	1.53	2.53	3.02	92.14	8.91
PAB	1.69	3.46	4.81	95.03	6.45
DTUA	1.80	2.87	2.52	91.51	7.38
PAC	1.63	2.92	2.89	94.62	8.01
AHDTRA	3.21	4.15	4.11	92.78	7.49
AHTRA	1.45	2.38	2.91	96.16	5.71
DPA	2.65	3.10	3.37	93.42	8.05
PA	1.42	2.46	3.64	90.25	9.42
DEA	2.28	3.98	4.25	91.06	8.76

## Supplementary Materials

Supplementary Materials are available online. Supplementary Table S1: Identification of triterpene compounds in the natural and fermented parts of *Poria cocos* using HPLC-QTOF-MS/MS.
