# Supplementary material for: Insights into Triterpene Acids in Fermented Mycelia of Edible Fungus Poria cocos by a Comparative Study
Source: Molecules. 2019 Apr 4;24(7):1331. doi: 10.3390/molecules24071331 (PMC6479485; doi:10.3390/molecules24071331)
Supplement: Supplementary file 1 [file molecules-24-01331-s001.pdf]

Supplementary Table S1

Identification of triterpene compounds in the natural and fermented parts of *Poria cocos* using HPLC-QTOF-MS/MS

No [a]	t_R (min)	Precursor (m/z)	Mass (m/z)	Error (ppm)	MS/MS (m/z)	Formula	Identification
1	7.28	513.3229	514.3305	-2.09	513.3224;495.3069;439.2845	$C_{31}H_{46}O_6$	Poricoic acid F
2	7.65	499.3070	500.3147	-1.74	499.3417;481.3343;427.2849; 349.2531	$C_{30}H_{44}O_6$	Poricoic acid E
3	8.41	499.3070	500.3508	-1.17	499.3417;481.3343;405.2818;389.2661; 97.0658	$C_{31}H_{48}O_5$	Poricoic acid H isomer
4	8.63	513.3222	514.3298	-0.73	513.3224;495.3103;439.2850;421.2754	$C_{31}H_{46}O_6$	Poricoic acid D
5	9.06	513.3222	514.3295	-0.20	513.3204;495.3041;439.2969;421.2718	$C_{31}H_{46}O_6$	Poricoic acid D
6	9.83	485.3275	486.3346	-0.11	485.3295;467.3188;441.3442;439.3192;407.2945;375 .2642	$C_{30}H_{46}O_5$	Poricoic acid G
7	10.98	499.3436	500.3509	-1.38	499.3416;481.3313;455.3554;437.3392;21.3084;409. 2810;193.1603;97.0657	$C_{31}H_{48}O_5$	Poricoic acid H
8	11.87	497.3286	498.3372	6.44	497.3285;453.3351;423.2968	$C_{31}H_{46}O_5$	25-Hydroxyporicoic acid C
9	13.74	513.3599	514.3664	-1.17	513.3263;469.3342;453.2933;425.2707	$C_{32}H_{50}O_5$	Poricoic acid HM
10	14.67	497.3283	498.3368	-4.56	497.3285;455.3552;437.3430;409.3135	$C_{31}H_{46}O_5$	Poricoic acid BM
11	15.44	469.337	470.3409	-0.02	469.3347;453.3451;407.3258;309.1855;291.2220	$C_{30}H_{46}O_4$	3β,16α-Dihydroxylanosta-7,9(11) , 24-trien-21-oic acid
12	16.16	467.3180	468.3255	-3.37	407.2960;391.2635;375.2407;321.2227;255.1381;227 .1500;179.1477;71.0150	$C_{30}H_{44}O_4$	16-Deoxyporicoic acid B
13	17.84	469.3334	470.3408	0.32	469.3311;407.2964;423.3253;393.2792;375.2671	$C_{30}H_{46}O_4$	16α-Hydroxydehydrotrametenoli c acid
14	18.86	471.3488	472.3563	-2.16	409.3115;337.2535;275.2020;245.1907;207.1766;137 .0988; 97.0668	$C_{30}H_{48}O_4$	16α-Hydroxytrametenolic acid
15	19.50	483.3129	484.3203	-0.19	483.3108;465.2995;409.2740;391.2641;365.2832	$C_{30}H_{44}O_5$	Poricoic acid B
16	21.32	483.3467	484.3565	0.18	483.3471;437.3432;421.3128;391.2941;311.2039;97. 0661	$C_{31}H_{48}O_4$	Dehydrotumulosic acid
17	21.98	497.3286	498.3368	-4.54	453.3384;423.2907;397.2740;211.1484;71.0154	$C_{31}H_{46}O_5$	3-Oxo-16α,25-dihydroxy-Lanost a-7,9(11),24(31)-trien-21-oic acid
18	22.55	497.3284	498.3357	0.08	497.3256;479.3141;453.3358;423.2888;379.2997;211 .1549;73.0322;61.9908	$C_{31}H_{46}O_5$	Poricoic acid A
19	22.58	485.3308	486.3381	-7.42	485.3640;467.3478;423.3276;337.2547;97.0667	$C_{31}H_{46}O_5$	Tumulosic acid

20	23.80	469.3339	470.3441	-0.23	407.2907;375.2695;273.1857;179.1426; 97.0663	$C_{30}H_{46}O_4$	3β,16α-Dihydroxylanosta-7,9(11), 24-trien-21-oic acid
21	24.74	543.3709	544.3779	-2.79	467.3524;337.2531;221.1909;137.0964;75.0094	$C_{33}H_{52}O_6$	25-Hydroxypachymic acid
22	26.56	481.3336	482.3410	-0.35	481.3318;463.3230;435.3262;419.3022;405.2794;387.2673;311.2010;97.0655	$C_{31}H_{46}O_4$	Polyporenic acid C
23	27.50	483.3491	484.3565	-2.46	421.3130;389.2822;337.2527;273.1873;193.1594;97.0662	$C_{31}H_{48}O_4$	16α-Hydroxyeburiconic acid
24	33.47	511.3441	512.3512	0.53	511.3416;493.3261;449.3133;389.2998;369.2028;59.0152	$C_{32}H_{48}O_5$	3-O-acetyl-16α-hydroxy-dehydrotrametenolic acid
25	35.08	513.3587	514.3675	-3.28	513.3575;483.3116;451.3206;391.2995;207.1741;179.1430;59.0153	$C_{32}H_{50}O_5$	3-O-acetyl-16α-hydroxy-trametenolic acid
26	36.41	525.3604	526.3674	-2.97	525.3604;507.3495;481.3270;465.3300;59.0154	$C_{33}H_{50}O_5$	Dehydropachymic acid
27	37.75	527.3741	528.3833	-3.39	527.3741;497.3233;465.3373;405.3169;221.1958;59.0152	$C_{33}H_{52}O_5$	Pachymic acid
28	40.86	453.3386	454.3460	-2.87	453.3386;435.3262;423.0840;71.2583;337.2547;323.2377; 309.2204; 97.0659	$C_{30}H_{46}O_3$	Dehydrotrametenolic acid
29	42.19	455.3539	156.3612	-1.96	425.3047;373.2741;339.2681;295.2078;141.0890	$C_{30}H_{48}O_3$	Trametenolic acid
30	43.10	467.3484	468.3614	-2.23	467.3484;437.3045;405.3093;371.2584;337.2523;323.2377; 97.0681	$C_{31}H_{48}O_3$	Dehydroeburicoic acid
31	44.37	469.3681	470.3752	1.60	469.3654;439.3154;373.2816;339.2669;97.0613	$C_{31}H_{50}O_3$	Eburicoic acid

[a], the No. correspond to the peak No. in figure 2.
